# Deep learning radiomic analysis of DCE-MRI combined with clinical characteristics predicts pathological complete response to neoadjuvant chemotherapy in breast cancer

**DOI:** 10.3389/fonc.2022.1041142

**Published:** 2023-01-05

**Authors:** Yuting Li, Yaheng Fan, Dinghua Xu, Yan Li, Zhangnan Zhong, Haoyu Pan, Bingsheng Huang, Xiaotong Xie, Yang Yang, Bihua Liu

**Affiliations:** ^1^ The First Clinical Medical College, Guangdong Medical University, Zhanjiang, China; ^2^ Department of Radiology, Dongguan People’s Hospital, Dongguan, China; ^3^ Medical Artificial Intelligence Lab, School of Biomedical Engineering, Health Science Center, Shenzhen University, Shenzhen, China; ^4^ Department of Radiology, Affiliated Hospital of Guangdong Medical University, Zhanjiang, China; ^5^ Department of Minimally Invasive Interventional Radiology, Guangzhou Panyu Central Hospital, Guangzhou, China; ^6^ Department of Radiology, Suining Central Hospital, Suining, China

**Keywords:** dynamic contrast-enhanced magnetic resonance imaging, breast cancer, pathological complete response, radiomics, deep learning

## Abstract

**Objective:**

The aim of this study was to develop and validate a deep learning-based radiomic (DLR) model combined with clinical characteristics for predicting pathological complete response (pCR) to neoadjuvant chemotherapy (NAC) in breast cancer. For early prediction of pCR, the DLR model was based on pre-treatment and early treatment dynamic contrast-enhanced magnetic resonance imaging (DCE-MRI) data.

**Materials and methods:**

This retrospective study included 95 women (mean age, 48.1 years; range, 29–77 years) who underwent DCE-MRI before (pre-treatment) and after two or three cycles of NAC (early treatment) from 2018 to 2021. The patients in this study were randomly divided into a training cohort (n=67) and a validation cohort (n=28) at a ratio of 7:3. Deep learning and handcrafted features were extracted from pre- and early treatment DCE-MRI contoured lesions. These features contribute to the construction of radiomic signature RS1 and RS2 representing information from different periods. Mutual information and least absolute shrinkage and selection operator regression were used for feature selection. A combined model was then developed based on the DCE-MRI features and clinical characteristics. The performance of the models was assessed using the area under the receiver operating characteristic curve (AUC) and compared using the DeLong test.

**Results:**

The overall pCR rate was 25.3% (24/95). One radiomic feature and three deep learning features in RS1, five radiomic features and 11 deep learning features in RS2, and five clinical characteristics remained in the feature selection. The performance of the DLR model combining pre- and early treatment information (AUC=0.900) was better than that of RS1 (AUC=0.644, P=0.068) and slightly higher that of RS2 (AUC=0.888, P=0.604) in the validation cohort. The combined model including pre- and early treatment information and clinical characteristics showed the best ability with an AUC of 0.925 in the validation cohort.

**Conclusion:**

The combined model integrating pre-treatment, early treatment DCE-MRI data, and clinical characteristics showed good performance in predicting pCR to NAC in patients with breast cancer. Early treatment DCE-MRI and clinical characteristics may play an important role in evaluating the outcomes of NAC by predicting pCR.

## 1 Introduction

The global cancer statistics 2020 reported that breast cancer has become the first cause of cancer worldwide, with over 2.26 million new cases (1). With the number of patients with breast cancer increasing annually, comprehensive breast cancer treatment has become increasingly crucial. Neoadjuvant chemotherapy (NAC) for breast cancer is a systemic chemotherapy conducted before surgery or radiotherapy and is an essential component of the overall treatment for breast cancer. Its main purpose is to decrease tumor load before surgery, reduce tumor stage, and convert inoperable tumors into operable ones. As a result, female patients with breast cancer who require total mastectomy may manage to preserve their breasts and improve their overall treatment outcomes and their quality of life (2). Furthermore, studies have shown a strong association between pathological complete response (pCR), overall survival, and disease-free survival (3). Therefore, early prediction of pCR has become the focus of clinical attention.

Numerous studies have proposed various methods, including mammography, digital breast tomography, ultrasonography, and magnetic resonance imaging (MRI), to assess the response to NAC in patients with breast cancer. MRI is effective in predicting pCR after the completion of NAC (4), and the dynamic contrast-enhanced MRI (DCE-MRI) technology is more reliable than other methods. In addition, a growing body of research has shown that DCE-MRI can reflect tumor microvascular perfusion and vascular permeability, providing the possibility of quantitative evaluation and is thus helpful in predicting the final outcome after completion of NAC for breast cancer (5, 6).

Radiomics was first proposed by Lambin (7) and involves extracting massive features from segmented medical images. By transforming medical images into mineable data for quantitative and qualitative analyses of tumor heterogeneity, radiomics is widely used to provide effective references for the management of various oncological diseases, particularly in differential diagnosis, subtype analysis, treatment option selection, prognosis evaluation, and efficacy assessment (8–10). Moreover, radiomics can be combined with clinical information, histopathological and molecular features, and multiple imaging features to provide more comprehensive information. Previous studies have shown that radiomic features obtained using DCE-MRI have good predictive ability for pCR after the completion of NAC in breast cancer (11). With the rapid development of deep learning and radiomic theories, a new technology that combines information from both deep learning and radiomic features has emerged. The radiomic method adopting deep learning-based radiomic (DLR) features has shown the first advanced performance in medical image analysis (12). Liang et al. (13) constructed a DLR nomogram to evaluate whether there were metastatic lesions in other organs in patients with soft tissue sarcoma before surgery. In the external validation cohort, the DLR nomogram model demonstrated better predictive power than either the radiomic or clinical model, with areas under the receiver operating characteristic curve (AUCs) of 0.833, 0.799, and 0.664, respectively. Li et al. (14) established a model for predicting the prognosis of stage II colorectal cancer by combining the deep learning and radiomic features of primary lesions and peripheral lymph nodes based on computed tomography images. The AUCs of disease-free survival and total survival of the model increased to 0.76 and 0.91, respectively.

Recent studies (15) have used DLR based on DCE-MRI to predict pCR to NAC in breast cancer, but the predictive power of the models was found to be unsatisfactory. In addition, models that comprehensively incorporate pre-treatment and early treatment DCE-MRI data, as well as clinical information, are almost non-existent. As the pre-treatment image features and clinical features are related to the characteristics of the primary tumor, the post-treatment images can reflect the tumor response to NAC drugs. Here, we hypothesized that improved performance could be achieved by constructing a model combining these three elements. As a result, we retrospectively included women with breast cancer who received NAC and obtained radiomic and deep learning semantic segmentation features based on pre-treatment and early treatment DCE-MRI data. We aimed to develop a model combined multi-period images information with clinical characteristics to predict pCR in female patients with breast cancer treated with NAC.

## 2 Materials and methods

### 2.1 Patient population

This retrospective study was conducted in accordance with the tenets of the Declaration of Helsinki and was approved by the ethics committee of Dongguan People’s Hospital, with the requirement for informed consent waived.

**Figure 1:**
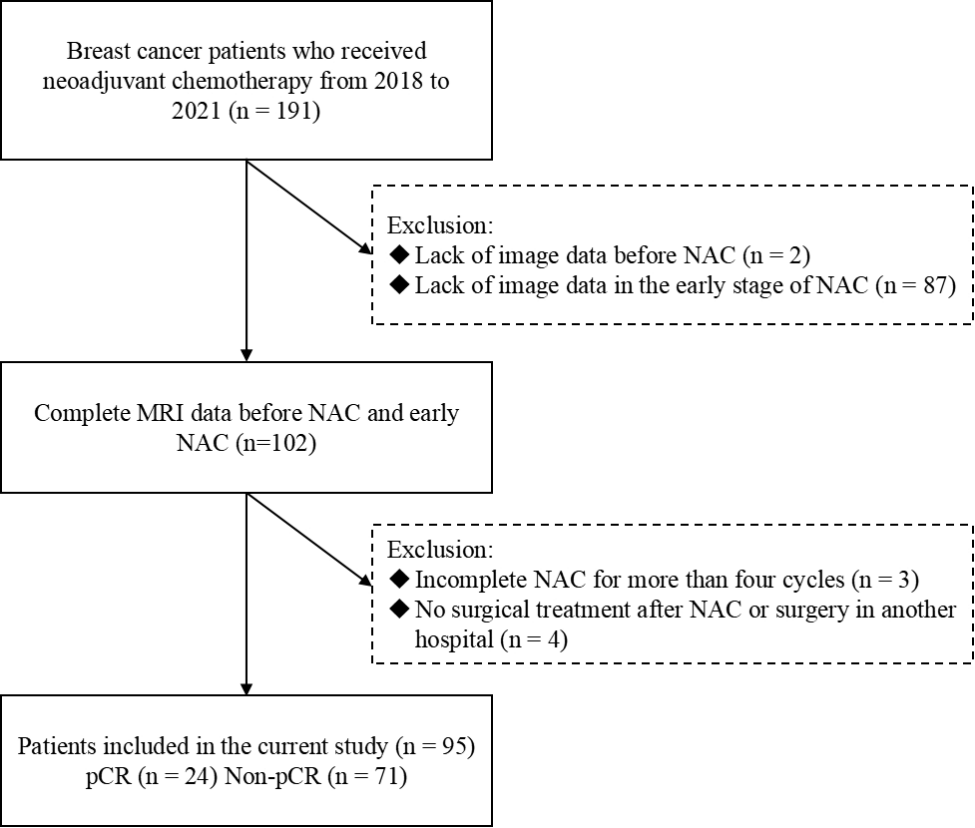
Flowchart of patient enrollment in the study.

Ninety-five patients with breast cancer who underwent NAC and breast DCE-MRI from 2018 to 2021 were enrolled in the study. The following criteria were required for inclusion: (i) primary breast cancer confirmed using core needle biopsy before the start of the treatment; (ii) availability of pre-treatment and post-treatment histopathologic information; and (iii) absence of any treatment before NAC. Patients meeting the following criteria were excluded: (i) lack of pre-treatment or early treatment DCE-MRI data; (ii) incomplete NAC for more than four cycles; and (iii) absence of surgical treatment in our hospital or having received surgical treatment outside our hospital after NAC. The study population enrollment pathway is shown in **Figure 1**.

To guarantee improved generalization performance, the dataset was divided into training and validation cohorts (70/30 divisions). The training cohort contained 67 patients (16 pCR, 51 non-pCR; mean age 47.8 years), and the validation cohort contained 28 patients (8 pCR, 20 non-pCR; mean age 48.9 years).

By reviewing medical records, clinically relevant information, including age, body mass index, menstrual status, NAC cycle, NAC regimen, genetic testing results, molecular typing, and clinical staging, was obtained from the patients with breast cancer. All patients obtained NAC (more than four cycles) and underwent surgical resection. The following NAC regimens were included in this study: (i) 15 cases of EC-T (epirubicin, cyclophosphamide, sequential docetaxel); (ii) 13 cases of AC-T (doxorubicin, cyclophosphamide, sequential docetaxel); (iii) 7 cases of EC-TH (epirubicin, cyclophosphamide, sequential docetaxel, and trastuzumab); (iv) 5 cases of EC-THP (epirubicin, cyclophosphamide, sequential docetaxel, trastuzumab, and pertuzumab); (v) 4 cases of AC-THP (doxorubicin, cyclophosphamide, sequential docetaxel, trastuzumab, and pertuzumab); (vi) 7 cases of TA (docetaxel, doxorubicin); (vii) 21 cases of TEC (docetaxel, epirubicin, cyclophosphamide); (viii) 11 cases of TAC (docetaxel, doxorubicin, cyclophosphamide); (ix) 8 cases of TCH (docetaxel, carboplatin, trastuzumab); and (x) 4 cases of TCHP (docetaxel, carboplatin, trastuzumab, and pertuzumab).

Immunohistochemical (IHC) results, including the expression of Ki67, progesterone receptor (PR), estrogen receptor (ER), and human epidermal growth factor receptor 2 (HER2), were obtained from a lesion biopsy conducted before NAC. According to the immunohistochemical index evaluation criteria (16), ER and PR positivity was defined as ≥1% of tumor cells with positive nuclear staining. Tumors with IHC staining of 3 were considered HER2 positive. IHC 2+ tumors require further confirmation *via* molecular testing (*in situ* hybridization (17) testing). The ISH-amplified results were determined to be HER2 positive. The cutoff value for Ki67 was 14% (16). The molecular subtype of the tumor was defined as follows (18): patients with ER and/or PR (+), HER2 (−), and Ki67<14% were grouped into luminal A; patients with ER and/or PR (+), HER2 (+) or ER, and/or PR (+), HER2 (−), and Ki-67≥14% were grouped into luminal B; patients with ER and PR (−) and HER2 (+) were grouped into the HER2 overexpression type; and patients with ER and PR (−) and HER-2 (−) were grouped into the triple-negative type.

No remaining cancer in the breast and axillary lymph nodes confirmed using lesion biopsy of the surgical specimen (residual ductal carcinoma *in situ* could be acceptable) was defined as pCR (19).

### 2.2 MRI acquisition

DCE-MRI data of the female patients with breast cancer were retrospectively collected. These examination data were generated when the patients underwent MRI before NAC and after two or three cycles of NAC. Patients were scanned using a 3.0-T scanner (Skyra, SIEMENS) with a dedicated 16-channel phased-array breast coil.

All DCE-MRI examinations were carried out using fast, low-angle shot fat-suppressed three-dimensional spoiled gradient-echo T1-weighted imaging. The following acquisition settings were applied: repetition time, 4.78 ms; echo time, 1.77 ms; matrix size, 256×256; resolution, 0.75 mm; flip angle, 10 degrees; field of view, 320 mm×320 mm; slice thickness, 1.70 mm; and slice gap, 50% of slice thickness. The contrast agent gadopentetate dimeglumine (produced by Hengrui Company, Jiangsu, China) at a dose of 0.15 mmol/kg was injected through the median elbow vein at a rate of 3–4 ml/s, followed by flushing 15 ml of saline. The contrast agent was injected at the end of the first scanning phase, and six phases were scanned, each lasting 72 s.

### 2.3 Region of interest segmentation

Two experienced radiologists (radiologist one who had worked in the areas of breast disease diagnosis for three years, and radiologist two who had worked in the areas of breast disease diagnosis for ten years) were invited. They were unaware of the pathology results and used the ITK-SNAP software (http://www.itksnap.org/) to delineate a region of interest layer by layer along the tumor margin on DCE-MRI images. As tumor enhancement was obvious in the third scanning phase, we chose to sketch the enhanced high-signal area in this image phase and attempted to sketch the burr at the edge of the tumor as completely as possible.

### 2.4 Feature extraction and selection

We used the pyradiomics toolkit (https://pyradiomics.readthedocs.io/en/v3.0.1/) to extract 1130 handcrafted radiomic features automatically. To speed up the network convergence and improve generalization, we resampled the image at a uniform scale and performed min-max normalization on the extracted features. As a result, 512 deep-learning semantic segmentation features were extracted based on the UCTransNet network structure (20), which was a semantic segmentation network based on U-Net and Transformer. The semantic segmentation network was first trained by using the segmentation dataset (70% of the training dataset in the classification task) to capture the characteristics of the lesions. Secondly, semantic features are extracted from the segmentation dataset and then used to construct the feature library for the feature similarity adaptation. We used K-means clustering to divide the features of each patient into two clusters. Afterward, we calculate the similarity between the two clusters and the feature library to select the optimal combination of effective features. The relevant information of the feature extraction process is described in **Supplementary Material S1**. Interclass correlation coefficients (ICCs) were employed to assess the intra- and inter-observer consistency of the feature extraction, and an ICC >0.80 was considered acceptable.

To ensure that the features most relevant to the prediction of pCR were retained, we adopted a coarse-to-fine feature selection method. The first step was to use the mutual information feature selection method in the scikit-learn toolkit (https://www.scikit-learn.org/) for initial screening. In the second step, the least absolute shrinkage and selection operator (LASSO) method was introduced to screen features obtained from the initial screening. LASSO involves the parameter λ in controlling the number of selected features. We used the 10-fold cross-validation method during the training process to select the optimal parameter λ to obtain the optimal number of features and to avoid overfitting. The relevant information of the feature selection process is described in **Supplementary Material S2**. Finally, from the pre-treatment DCE-MRI data, we retained one radiomic feature and three deep-learning features as RS1. From the DCE-MRI at early treatment data, we retained five radiomic features and 11 deep learning features as RS2. More details on these features are provided in the **Supplementary Materials** in **Table S1**, and the clinical features included in the model comprised expression of ER, PR, and HER2; clinical N stage; and clinical TNM stage.

### 2.5 Model development and validation

**Figure 2:**
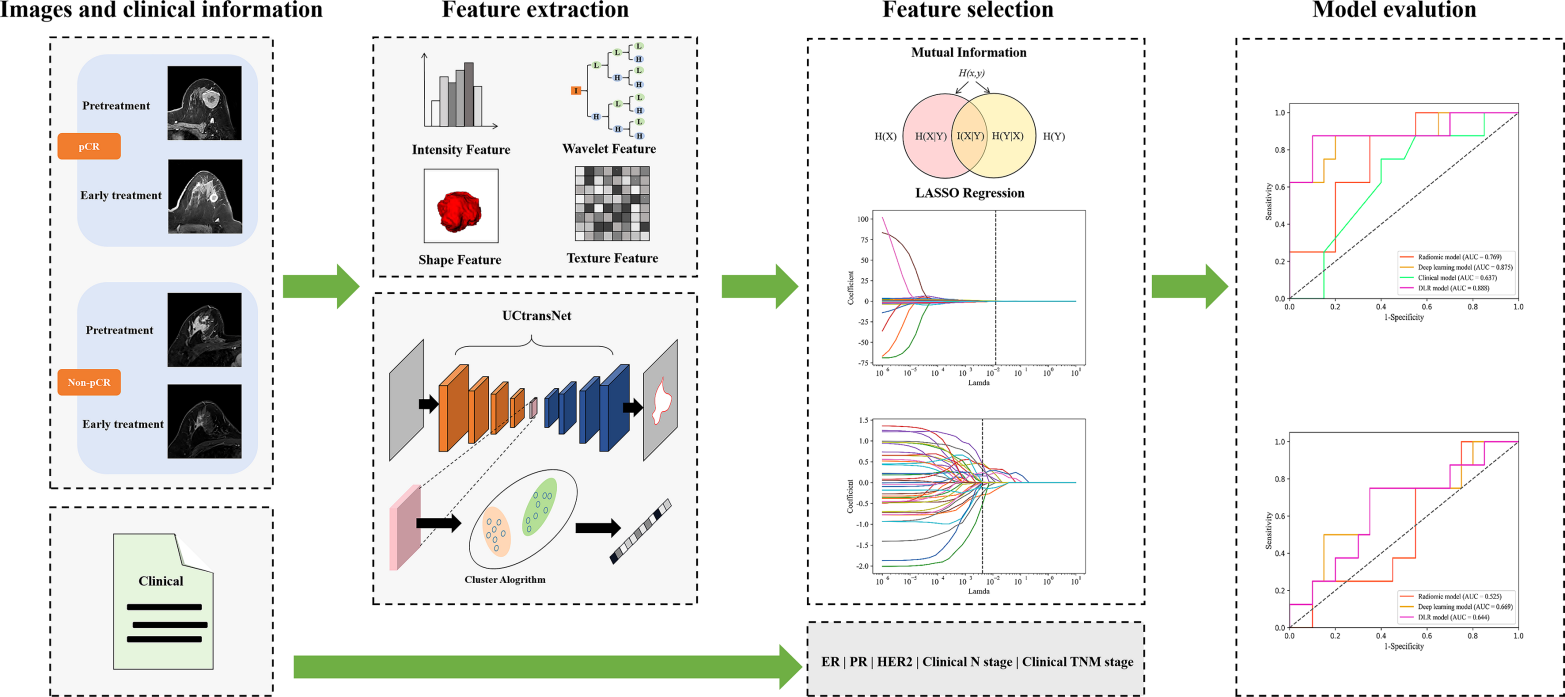
The workflow for building DLR models.

The study group was randomly divided into training and validation cohorts at a ratio of 7:3. The model parameters were then optimized in the training cohort using a five-fold cross-validation scheme. In the cross-validation process, the four-fold data were used as the training dataset, and the one-fold data were used as the validation dataset, which was repeated five times, and then the predicted probability of the five-fold validation data was used as a whole to evaluate the performance of the model. During independent testing, we evaluated model performance with the mean of the predicted probabilities of all models produced by cross-validation. A flowchart of the DLR method is shown in **Figure 2**.

Six models were built to explore the performance differences of models constructed with various features over different periods. First, we constructed the corresponding models using traditional radiomics, deep learning and clinical features, respectively. Second, we combined the radiomics and deep learning features and feed them into the classifier to construct the DLR model. Then, we developed the models that composed of clinical information and single-epoch image features. To completely use all information from the traditional radiomic features, deep-learning semantic segmentation features and clinical characteristics, we spliced these data to construct a combined model. The prediction results of the two models (constructed from the three features of pre-treatment and early treatment, respectively) were integrated. The output probability of the two models averaged the final probability of each patient. Finally, a logistic regression classifier was used to build our model from the selected best radiomic features, deep learning features, and clinical characteristics.

### 2.6 Performance of the model

The calibration curve and Hosmer–Lemeshow test were adopted to calibrate the model. The AUC was used to evaluate the discrimination ability of the model. The sensitivity, specificity, positive predictive value, negative predictive value, and accuracy of the model were calculated according to the optimal cut-off value that maximized the Youden index. The AUC values of the various models were compared using the Delong test.

### 2.7 Statistical analysis

All statistical analyses in this study were performed using MedCalc software (V.20.0.19.7; 2011 MedCalc Software Bvba, Mariakerke, Belgium) and Python 3.6.12 (https://www.python.org). Differences in the menstruation status; PR, ER, HER2, and Ki-67 expression; molecular subtypes; and clinical T, N, and TNM stages were compared between the pCR and non-pCR groups using the chi-square test or Fisher’s exact test. The t-test or Mann–Whitney U test was used to compare differences in age and body mass index. The AUC values of the models were compared using the DeLong method. All statistical tests were two-sided, and the statistical significance level was set at P<0.05.

## 3 Results

### 3.1 Clinical characteristics

A total of 95 female patients (mean age, 48.1 years; range, 29–77 years) were included in this study. The baseline clinical characteristics of the patients are listed in **Table 1**. Twenty-four patients (25.3%) achieved pCR, and 71 patients (74.7%) did not achieve pCR. No significant differences were found in the menstruation status; ER, HER2, and Ki-67 expression; molecular subtype; and clinical T, N, and TNM stages between the pCR and non-pCR groups, except for the PR status (P=0.007).

**Table 1:** Patient characteristics.

Characteristics	Non-pCR (n=71)	pCR (n=24)	P value ^a^
**Age, mean ± SD, years**	48.6 ± 10.6	46.8 ± 9.4	0.467
**BMI, mean ± SD**	23.6 ± 3.1	24.5 ± 4.8	0.791
**Menstruation**			0.105
Premenopausal	40 (56.3)	18 (75.0)	
Postmenopausal	31 (34.7)	6 (25.0)	
**ER status**			0.111
Positive	56 (78.9)	15 (62.5)	
Negative	15 (21.1)	9 (38.5)	
**PR status**			**0.007**
Positive	58 (81.7)	13 (54.2)	
Negative	13 (18.3)	11 (45.8)	
**HER2 status**			0.069
Positive	21 (29.6)	12 (50.0)	
Negative	50 (70.4)	12 (50.0)	
**Ki-67 status**			0.383
≥14%	53 (74.6)	20 (83.3)	
<14%	18 (25.4)	4 (16.7)	
**Molecular subtypes**			0.184
Luminal A	7 (9.9)	1 (4.2)	
Luminal B	55 (77.4)	16 (66.7)	
HER2 overexpression	2 (2.8)	3 (12.5)	
Triple-negative	7 (9.9)	4 (16.6)	
**Clinical T stage**			0.340
T1-T2	49 (69.0)	19 (79.2)	
T3-T4	22 (31.0)	5 (20.8)	
**Clinical N stage**			0.381
N0-N1	59 (83.1)	18 (75.0)	
N2-N3	12 (16.9)	6 (25.0)	
**Clinical TNM stage**			0.682
I-II	41 (57.7)	15 (62.5)	
III-IV	30 (42.3)	9 (37.5)	

Unless otherwise specified, the data are the number of patients, with percentages in parentheses.

BMI, body mass index; ER, estrogen receptor; PR, progesterone receptor; HER2, human epidermal growth factor receptor-2; SD, standard deviation

**^a^** Comparison between pCR cohort and non-pCR cohort. Bold indicates p<0.05, the difference is statistically significant.

### 3.2 Reproducibility and interpretability of features

To investigate inter- and intra-observer feature reproducibility, 20 patients (10 with pCR and 10 without pCR) were randomly selected, and their regions of interest were reassessed by readers 1 and 2. A two-way random effects model was used to calculate ICCs to determine inter- and intra-observer reliability. The median ICCs for intra- and inter-observer consistency evaluation of radiomic features extracted before chemotherapy were 0.966 (interquartile range [IQR], 0.907–0.987) and 0.969 (IQR, 0.911–0.989), respectively. The median ICCs for intra- and inter-observer consistency evaluation of radiomic features extracted from two to three cycles of chemotherapy were 0.953 (IQR, 0.892–0.978) and 0.952 (IQR, 0.886–0.978), respectively.

**Figure 3:**
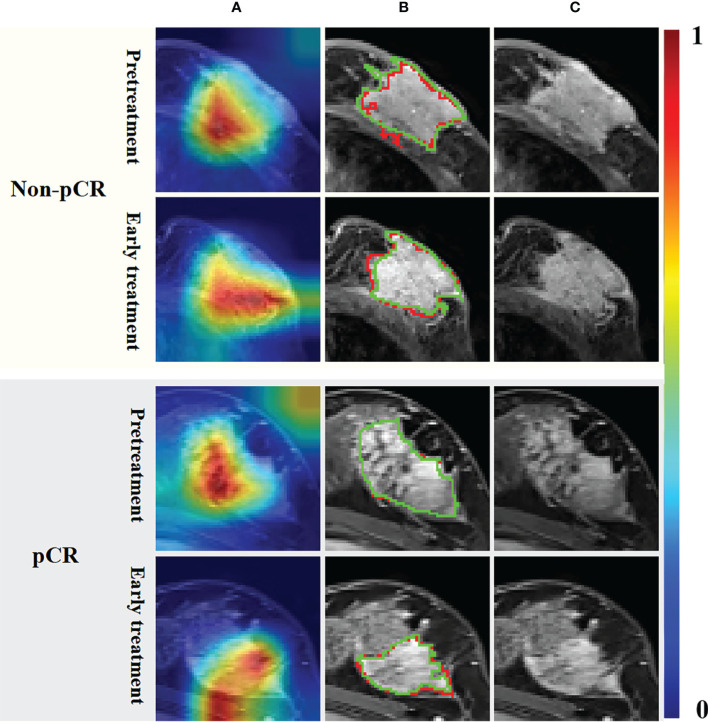
Grad-Cam heat maps **(A)** calculated by the UCtransNet network, segmentation results **(B)** and DCE-MRI **(C)**. The red line in the segmentation result represents the prediction result, and the green line represents the ground truth. The red and yellow regions in the heat maps represent areas with higher activation, while the blue and green regions represent lower activation.

As shown in **Figure 3**, lesions in patients with pCR did not invade adjacent muscle tissue, but those in patients with non-pCR did. The Grad-Cam (21) heat maps showed that the adjacent regions between breast cancer lesions and adjacent tissues were activated, indicating that our model correctly identified the target area and the extracted features effectively reflected the relevant information of pCR. This is consistent with the current literature reports (22) that tumor staging is related to pCR rate. The segmentation results of the DL model with an average DSC of 0.77 for pretreatment breast cancer lesion segmentation and 0.74 for early treatment. We think that the CNN network achieves appropriate segmentation performance, thus extracting semantic information about segmented regions from the hierarchical convolutional layers of the network, and good classification performance can prove that the extracted features are effective.

### 3.3 Comparison of the radiomic, deep learning, DLR, and clinical models based on the pre- or early-treatment images

**Figure 4:**
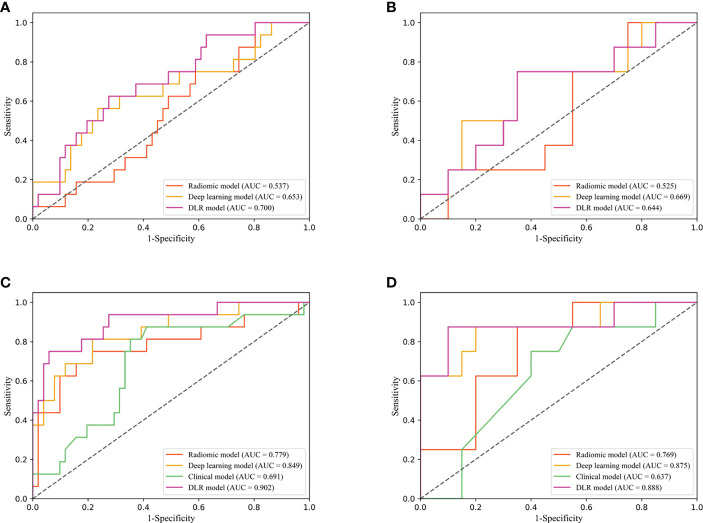
Diagnostic performance of DLR model in the training and validation cohorts. Receiver operating characteristic (ROC) curves for performance comparison of radiomics, deep learning and DLR model based on the pretreatment DCE-MRI in the training **(A)** and validation cohorts **(B)**. ROC curves for performance comparison of radiomics, deep learning, clinical model and DLR model based on the early treatment DCE-MRI in the training **(C)** and validation cohorts **(D)**.

The predictive performance comparison of the radiomic, deep learning, and DLR models based on the DCE-MRI data at pre-treatment is presented in **Table 2** and **Figures 4A, B**. In the training cohort, the DLR model (AUC=0.700) performed better than the radiomic model (AUC=0.537, P=0.046), but no difference was found between the DLR and deep learning models (AUC=0.653, P=0.332). Significant differences were not observed among the DLR (AUC=0.644), radiomic (AUC=0.525, P=0.333), and deep learning (AUC=0.669, P=0.577) models in the validation cohort. The predictive performance comparison of the radiomic, deep learning, and DLR models at early treatment is presented in **Table 3** and **Figures 4C, D**. The DLR model achieved an AUC of 0.902 and performed better than the radiomics model with an AUC of 0.779 (P=0.050) and the clinical model with an AUC of 0.691 (P=0.023). A significant difference was not found between the DLR and deep learning models (AUC=0.849, P=0.141) for the training cohort, and the same trend was observed for the validation cohort.

**Table 2:** Performance of pre-treatment image-based radiomics, deep learning and DLR models.

Variable	AUC (95% CI)	Accuracy	Sensitivity	Specificity	PPV	NPV	P value ^a^
Training cohort (n = 67)							
Radiomic model	0.537 (0.417-0.656)	0.388	1.000	0.196	0.281	1.000	.636
Deep learning model	0.653 (0.527-0.765)	0.716	0.563	0.765	0.429	0.696	.072
DLR model	**0.700 (0.575-0.806)**	0.701	0.625	0.725	0.417	0.861	**.007**
Validation cohort (n = 28)							
Radiomic model	0.525 (0.329-0.716)	0.464	1.000	0.250	0.348	1.000	.836
Deep learning model	**0.669 (0.467-0.834)**	0.679	0.750	0.650	0.462	0.867	.167
DLR model	0.644 (0.442-0.814)	0.679	0.750	0.650	0.462	0.867	.236

CI, confidence interval; AUC, area under the receiver operating characteristic curve; PPV, positive predictive value; NPV, negative predictive value; DLR, deep learning radiomic.

a P value is the significance level of comparison of AUC with that of random case (AUC = 0.5). Bold indicates p<0.05, the difference is statistically significant.

**Table 3:** Performance of early treatment image-based radiomics, deep learning, clinical and DLR models.

Variable	AUC (95% CI)	Accuracy	Sensitivity	Specificity	PPV	NPV	P value ^a^
Training cohort (n = 67)							
Radiomic model	0.779 (0.661-0.872)	0.776	0.750	0.784	0.522	0.909	**<.001**
Deep learning model	0.849 (0.741-0.925)	0.791	0.813	0.784	0.542	0.930	**<.001**
Clinical model	0.691 (0.566-0.798)	0.657	0.875	0.588	0.400	0.938	**.012**
DLR model	**0.902 (0.805-0.961)**	0.896	0.750	0.941	0.800	0.923	**<.001**
Validation cohort (n = 28)							
Radiomic model	0.769 (0.572-0.906)	0.714	0.875	0.650	0.500	0.929	**.004**
Deep learning model	0.875 (0.695-0.969)	0.821	0.875	0.800	0.636	0.821	**<.001**
Clinical model	0.637 (0.435-0.809)	0.643	0.750	0.600	0.429	0.857	.220
DLR model	**0.888 (0.711-0.975)**	0.893	0.875	0.900	0.778	0.947	**<.001**

CI, confidence interval; AUC, area under the receiver operating characteristic curve; PPV, positive predictive value; NPV, negative predictive value; DLR, deep learning radiomic.

a P value is the significance level of comparison of AUC with that of random case (AUC = 0.5). Bold indicates p<0.05, the difference is statistically significant.

### 3.4 Combined model combining multi-period images and clinical information

**Figure 5:**
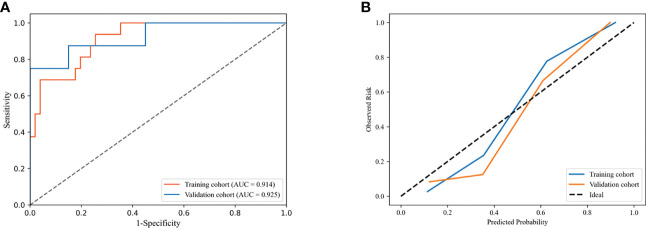
Receiver operating characteristic (ROC) curves **(A)** and calibration curves **(B)** of combined model in the training and validation cohorts.

The combined model integrated multi-period images with clinical information. The AUC of the model according to the DeLong test was not significantly different between the training and validation cohorts (P=0.878), indicating that our model showed good validity and stability (**Figure 5A**). The calibration curve (**Figure 5B**) and the chi-squared values of the Hosmer–Lemeshow test of the training (χ^2 =^ 4.402, P=0.819) and validation (χ^2 =^ 6.087, P=0.638) cohorts indicated that the model fit the data well.

**Figure 6:**
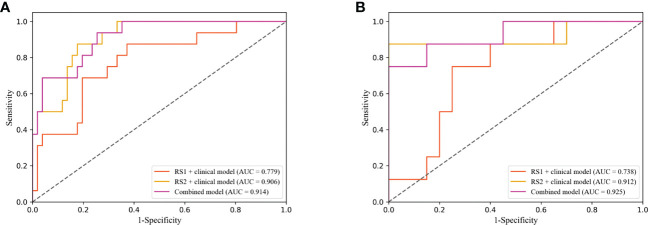
Receiver operating characteristic (ROC) curves of combined model combining multi-period image with clinical information and the combined model combining single-period image with clinical information in training **(A)** and validation cohorts **(B)**.

The performance of the DLR model combining multi-period images in predicting pCR in the training and validation cohorts was AUC of 0.908 and 0.900, respectively. The combined model combining multi-period images with clinical information showed better performance than the model combining single-period images with clinical information (**Table 4** **;** **Figures 6A, B**). In the training cohort (**Figure 6A**), the predicted performance with an AUC of 0.914 for the model combining multi-period image information and clinical information. The AUCs of pCR predicted using the combined model with RS1 and clinical information only and the combined model with RS2 and clinical information only were 0.779 and 0.906 (P=0.030 and 0.767), respectively. For the validation cohort (**Figure 6B**), the AUC of the combined model was 0.925. Its performance somewhat improved compared with that of the combined model with RS1 and clinical information only, with an AUC of 0.738 (P=0.079), and that of the combined model with RS2 and clinical information only, with an AUC of 0.912 (P=0.769).

**Table 4:** Performance of combined model combining multi-period image with clinical information and the model combining single-period images with clinical information.

Variable	AUC (95% CI)	Accuracy	Sensitivity	Specificity	PPV	NPV	P value ^a^
Training cohort (n = 67)							
RS1 + Clinical model	0.779 (0.661-0.872)	0.686	0.875	0.627	0.424	0.941	**<.001**
RS2 + Clinical model	0.906 (0.809-0.963)	0.836	0.875	0.824	0.609	0.955	**<.001**
Combined model	**0.914 (0.820-0.969)**	0.791	0.938	0.745	0.536	0.974	**<.001**
Validation cohort (n = 28)							
RS1 + Clinical model	0.738 (0.538-0.884)	0.750	0.750	0.750	0.546	0.882	**.017**
RS2 + Clinical model	0.912 (0.743-0.986)	0.964	0.875	1.000	1.000	0.952	**<.001**
Combined model	**0.925 (0.760-0.990)**	0.928	0.750	1.000	1.000	0.927	**<.001**

CI, confidence interval; AUC, area under the receiver operating characteristic curve; PPV, positive predictive value; NPV, negative predictive value; RS1, pretreatment radiomic signature; RS2, early treatment radiomic signature

a P value is the significance level of comparison of AUC with that of random case (AUC = 0.5). Bold indicates p<0.05, the difference is statistically significant.

## Discussion

In the present study, we used pre-treatment and early treatment DCE-MRI data to evaluate tumor heterogeneity during NAC to predict pCR in female patients with breast cancer. A combined model that integrates multi-period image features and clinical information was developed. The results showed that the model yielded the highest AUCs of 0.914 and 0.925 in the training and validation cohorts, respectively. The good performance of the combined model showed that the combination of the model containing multi-period images and clinical characteristics can effectively predict the response to NAC before surgery and provide valuable information to assist doctors in making clinical decisions.

Radiomics has been successfully used to predict pCR after NAC in patients with breast cancer (23, 24). Eun et al. (25) used texture analysis for early prediction of pathological response in breast cancer, and achieved an AUC of 0.82 with mid-treatment contrast-enhanced T1-weighted MRI. They compared the difference of pretreatment and mid-treatment MRI images in texture features. However, a comprehensive analysis combining pre- and mid-treatment MRI features was not performed. In our study, we demonstrated that this combination improved the model performance. Fan et al. (26) fused molecular subtype information and radiomic features from pre- and early-treatment MRI images to predict breast cancer response to neoadjuvant chemotherapy, achieving an AUC of 0.809. In our study, we not only developed the traditional radiomics models, but also established the deep learning models. Our results showed that the deep learning method based on either pre-treatment or early treatment images was better than the traditional radiomics method. Peng et al. (15) also applied the deep learning method to predict the pathological response to NAC for breast cancer and found that its performance was better than that of the traditional radiomics method.

DLR has emerged in recent years. The DLR method showed good performance in predicting axillary lymph-node metastasis in early-stage breast cancer (27) and in identifying ocular adnexal lymphoma and idiopathic orbital inflammation (28). In addition, it has been successfully used in ultrasonography to predict the pCR of breast cancer to NAC (29, 30). Gu et al. (30) successfully applied DLR in ultrasound to predict the response to NAC in breast cancer patients in early treatment. The DLR models in these studies focused on using the deep learning classification network to automatically extract the high-dimensional features, however ignored the phenotypic features, such as shape and texture, which potentially reflect biologic properties like intra- and intertumor heterogeneities (31). In contrast, we applied a semantic segmentation network to extract deep learning features because this network structure can successfully extract useful information on tumor boundary, shape and texture (32). Therefore, the number of useful features in the segmentation network can be large even with relatively few training samples (33). Moreover, the segmentation results can be used as a visualization to provide some interpretability for the model. We further assumed that the handcrafted radiomic features and the deep learning radiomics feature could complement each other. Thus, we proposed a DLR model combining both deep learning features with traditional radiomics, and showed that the performance of DLR model was better than that of the deep learning and traditional radiomics models.

According to our results, our combined model’s performance was better than that of other models, which improved the ability of DCE-MRI to distinguish between pCR and non-pCR during NAC and compensated for the limitations of traditional strategies in predicting pCR. The major reason for the robustness of the combined model is the integration of pre-treatment and early treatment DCE-MRI data in the analysis process. This combination considered real-world clinical practice well and could provide meaningful guidance that somewhat benefits the patients. Notably, our results revealed that the performance of the model based on early treatment DCE-MRI data was better than that based on pre-treatment DCE-MRI data, proving the significant predictive value of early treatment DCE-MRI. Jiang et al. (29) used ultrasound images obtained before and after treatment to predict the pathological response of locally advanced breast cancer after NAC. Post-treatment images were found to reflect the present condition of the tumor after NAC, which was closer to the pathology after surgery. Therefore, the reliability of the model for predicting pCR can theoretically be improved by including post-treatment image features. The combination of clinical characteristics and imaging features allows for a more comprehensive description of breast tumors, and the performance of the model combining clinical characteristics with imaging features may be improved in some ways (34). Therefore, we included clinicopathological factors in the DLR model. According to the analysis of the LASSO algorithm, the ER, PR, and HER2 states and clinical N and clinical TNM stages differed from radiomics features and were incorporated into our model. The ER, PR, and HER2 states and clinical N stage have been proven to be associated with pCR after NAC in breast cancer (35–38), and the results showed that adding clinical information enables the DLR model to achieve greater predictive efficacy.

Our study also had several limitations. First, this was a single-center study with an inadequate sample size, which might have led to selection bias. Prior to future clinical application, we need to obtain more evidence from multiple centers to validate the model. Second, the study was retrospective in nature; thus, conducting a well-designed prospective study is necessary. Third, we only combined the image features and clinical characteristics in the study. Therefore, we will try more start-of-the-art methods to see if the performance of model can be further improved. Finally, only the tumor region features of the images were extracted, regardless of the model. To further refine the task, we need to obtain peritumoral tissues (39) before and during early treatment for comprehensive analysis.

In conclusion, based on pre-treatment and early treatment DCE-MRI images and clinical characteristics, we established a pCR prediction model using a DLR method that achieved good performance in the training and validation cohorts. The model can help clinicians evaluate whether the patient can reach pCR after NAC and can provide an effective diagnostic reference for accurate medical treatment of patients receiving NAC.

## Data availability statement

The raw data supporting the conclusions of this article will be made available by the authors, without undue reservation.

## Author contributions

BL, YY, and YTL conceived and designed the experiment. DX provided administrative support. YTL and YL collected and delineated images and collected clinical data on patients. YF performed the experimental design, analyzed the data, and prepared graphs. YTL and YF wrote the manuscript. BH, ZZ, HP, and XX assisted with manuscript writing and data analysis. All authors contributed to the article and approved the submitted version.
