# Supplementary material for: Deep learning radiomic analysis of DCE-MRI combined with clinical characteristics predicts pathological complete response to neoadjuvant chemotherapy in breast cancer
Source: Front Oncol. 2023 Jan 5;12:1041142. doi: 10.3389/fonc.2022.1041142 (PMC9850142; doi:10.3389/fonc.2022.1041142)
Supplement: Supplementary file 1 [file DataSheet_1.docx]

Supplementary Material

**Supplementary S1. Feature Extraction**

**Part 1. Radiomic Feature Extraction**

We analyzed the radiomic features by adopting the V3.0.1 version of Pyradiomics. The images were normalized with a normalization ratio of 50, and the BSpline interpolator method was used to resample all images to 0.8×0.8×3mm, which was the average spacing of training set. Radiomic features we extracted included first order statistics, shape-based (2D and 3D), Gray Level cooccurence matrix (GLCM), Gray Level Size Zone Matrix (GLSZM), Gray Level Run Length Matrix (GLRLM), Neighbouring Gray Tone Difference Matrix (NGTDM), Gray Level Dependence Matrix (GLDM). All the above features were extracted using the default settings. In addition, there are Laplacian of Gaussianfiltered (with Sigma values 1.0, 3.0, 5.0), wavelet-decomposition-based (using the coiflet 1 function). A specific description of the feature can be found in the Pyradiomics document. For each patient, we extracted 1130 features each from the pretreatment and early treatment DCE-MRI.

**Part 2. Deep Learning Feature Extraction**

As for the extraction of deep learning features, we also normalized the images and resampled to 0.8×0.8×3mm. Then cuted out the slices of their respective images within the range of slices and expanded them by 15 pixels according to the radiologist's notes (in the "region of interest" section). Finally, the cropped images were scaled to 64×64 pixels, which was used to input the segmentation network.

In the training phase, We employed the Adam optimizer to train our model, and also employed the combined cross entropy loss and dice loss as our loss function to train our network. Each mini-batch contains 12 image patches, the initial learning rate was set to 0.0001. We inputted the images into the trained segmentation network and extracted feature maps of the 4th down-sampled activation layer. Then, the feature maps would be operated through Global Average Pooling (GAP), and the output of GAP is the features each image. The feature library was the average of all features that the segmentation training set. We calculated the Euclidean distance between the average of the clustered features of the two clusters and the feature library, and assumed that the average of the cluster with the smaller distance was the effective feature of the patient.

The backbone network architecture chosen to extract image features is UCTransNet, which is a semantic segmentation network based on U-Net and Transformer. In this network, a channel converter (Channel Transformer, CTrans) is designed to replace the variant U-Net of skip connection in U-Net. It consists of two modules: CCT (Channel-wise Cross Fusion Transformer) for multi-scale encoder feature fusion and CCA (Channel-wise Cross Attention) for decoder feature fusion and enhanced CCT feature fusion. Because the upsampling phase adaptively utilizes all the downsampling phase information, the focus information lost due to downsampling can be retained. The convolutional neural network consists of four lower sampling layers and four upper sampling layers. All the downsampling features are fused and connected by transformer, thus the image is reconstructed, the model pays more attention to the global information, and the performance of the model is improved. Each lower sampling layer and upper sampling layer is comprises of 2 groups of grouped convolution, and a grouped convolution consists 3×3 convolution, BN layer and Relu layer in turn.

The network architecture of UCTransNet is implemented using Python pytroch (https:/pytorch.org/get-started/locally/) package.

# **Supplementary S2.** Feature Selection

We screened the radiomic features, deep learning features and clinical characteristics of tumors that were exracted during pretreatment and early treatment, and the details are described as follows:

1. For the radiomic features and deep learning features of tumors during pretreatment and early treatment, only the top 20 features most related to pCR status were retained by calculating the MI between features and pCR status.
2. In order to finally retain the most representative features, the features screened in the first step were further finely screened by LASSO, and the parameter lamda values of the LASSO screened RS1 and RS2 features were 0.0127 and 0.004, respectively.

After the above two steps, the radiomic and deep learning features were finally retained and would be further analyzed. Due to the small number of clinical characteristics, only LASSO method was used to screen them, and the retained clinical features would also be included in the analysis.

# Supplementary Table S1: **Input features of the radiomics and deep learning semantic segmentation.**

| Signatures | Features | Feature types | Weight | pCR  (mean ± SD) | Non-pCR  (mean ± SD) | *P* |
| --- | --- | --- | --- | --- | --- | --- |
| RS1 | wavelet-LHL_gldm_GrayLevelVariance | GLDM | -0.013 | 0.091 ± 0.066 | 0.185 ± 0.241 | **0.004** |
|  | DL111 | Deep learning | -0.042 | 0.304 ± 0.170 | 0.439 ± 0.198 | **0.004** |
|  | DL254 | Deep learning | 0.048 | 0.514 ± 0.274 | 0.497 ± 0.234 | 0.066 |
|  | DL411 | Deep learning | -0.387 | 0.420 ± 0.193 | 0.548 ± 0.216 | **0.012** |
| RS2 | wavelet-LLH_glszm_ZoneEntropy | GLSZM | -0.656 | 0.535 ± 0.256 | 0.759 ± 0.132 | **<0.001** |
|  | wavelet-LHL_glszm_LowGrayLevelZoneEmphasis | GLSZM | -0.163 | 0.269 ± 0.202 | 0.179 ± 0.206 | 0.068 |
|  | wavelet-HLL_glrlm_LongRunHighGrayLevelEmphasis | GLRLM | -0.019 | 0.111 ± 0.114 | 0.208 ± 0.187 | **0.019** |
|  | wavelet-HLH_glszm_GrayLevelNonUniformity | GLSZM | -0.007 | 0.057 ± 0.100 | 0.137 ± 0.173 | **0.007** |
|  | wavelet-HLH_gldm_DependenceNonUniformityNormalized | GLDM | 0.202 | 0.198 ± 0.255 | 0.097 ± 0.075 | 0.066 |
|  | DL23 | Deep learning | -0.110 | 0.476 ± 0.190 | 0.655 ± 0.197 | **<0.001** |
|  | DL66 | Deep learning | 0.331 | 0.509 ± 0.216 | 0.261 ± 0.165 | **<0.001** |
|  | DL90 | Deep learning | 0.219 | 0.483 ± 0.204 | 0.291 ± 0.163 | **<0.001** |
|  | DL137 | Deep learning | -0.017 | 0.306 ± 0.173 | 0.415 ± 0.175 | **0.009** |
|  | DL169 | Deep learning | -0.387 | 0.262 ± 0.137 | 0.309 ± 0.198 | 0.208 |
|  | DL227 | Deep learning | 0.121 | 0.721 ± 0.203 | 0.434 ± 0.226 | **<0.001** |
|  | DL305 | Deep learning | -0.525 | 0.371 ± 0.240 | 0.285 ± 0.162 | 0.050 |
|  | DL328 | Deep learning | 0.463 | 0.426 ± 0.257 | 0.284 ± 0.176 | **0.018** |
|  | DL341 | Deep learning | 0.098 | 0.608 ± 0.180 | 0.397 ± 0.170 | **<0.001** |
|  | DL388 | Deep learning | 0.342 | 0.514 ± 0.280 | 0.429 ± 0.173 | 0.172 |
|  | DL420 | Deep learning | -0.152 | 0.240 ± 0.220 | 0.443 ± 0.231 | **<0.001** |
